# Toxic effects of cadmium on tall fescue and different responses of the photosynthetic activities in the photosystem electron donor and acceptor sides

**DOI:** 10.1038/s41598-017-14718-w

**Published:** 2017-10-30

**Authors:** Meiyu Huang, Huihui Zhu, Jing Zhang, Diyong Tang, Xiaole Han, Liang Chen, Dongyun Du, Jun Yao, Ke Chen, Jie Sun

**Affiliations:** 1 College of Resources and Environmental Science, South-Central University for Nationalities, 182 Minyuan Road, Hongshan District, Wuhan, 430074 P.R. China; 2 Key Laboratory of Plant Germplasm Enhancement and Specialty Agriculture, Wuhan Botanical Garden, The Chinese Academy of Science, Lumo Street, Wuhan, 430074 P.R. China; 3 Key Laboratory of Catalysis and Materials Science of the State Ethnic Affairs Commission & Ministry of Education, Hubei Province, College of Resources and Environmental Science, South-Central University for Nationalities, Wuhan, 430074 P. R. China; 4 China University of Geosciences Beijing, School of Water Resources & Environment, Beijing, 100083 P.R. China; 5 Research Center of Human-Environment Relations, South-Central University for Nationalities, 182 Minyuan Road, Hongshan District, Wuhan, 430074 P.R. China

## Abstract

Tall fescue (*Festuca arundinacea Schreb*) is a turf grass species which is widely used for rhizoremediation of organic contaminants and shows notable prospects in heavy metal phytoremediation. In this study, different concentrations of cadmium ion (Cd^2+^) were applied to study toxic effects of Cd^2+^ and responses of tall fescue by soilless culture. Tall fescue showed comparable high tolerance to Cd^2+^ as Indian mustard (*Brassica juncea* L.). Additionally, the treatment with high concentration of Cd^2+^ leaded to decreased chlorophyll contents, production of reactive oxygen species (ROS) and lipid peroxidation, as well as damage of cell membrane, necrosis and apoptosis in tall fescue roots, and toxicity of Cd^2+^ on physiologic properties of tall fescue has been well discussed. Moreover, in photosystem II electron donor side, electron transport from oxygen evolution complex (OEC) to Yz residue of D1 protein was inhibited under high Cd^2+^ treatments, which may be due to the Cd^2+^ induced ROS production and the replacement of Ca^2+^ in the core of OEC. In electron acceptor side, electron transport efficiency from quinone B to photosystem I acceptors increased under high Cd^2+^ treatments, which may be an important response for plants against Cd^2+^ toxicity and its mechanism needs our further study.

## Introduction

Cadmium (Cd) is a dominant industrial and environmental pollutant in soils and it is widespread in environment especially in developing countries. It has deleterious effects on organisms within soils, associated vegetation cover as well as animals by food chain transport. In a certain concentration range, Cd shows greater toxicity to plants than the other heavy metals^1^. In plants, Cd could inhibit root growth, decrease chlorophyll content and photosynthesis, interfere with carbon metabolism, plant water status and nutrient uptake^2^. Through food chain and drinking water, it causes severe damage to a variety of organs including lung, liver, kidney, testis and placenta^3^.

Remover of Cd from soil environment has been an important issue. Phytoremediation is the use of plants to reduce concentrations or toxic effects of contaminants in the environments, and it is cost-effective, efficient, environmental friendly, and solar-driven technology with good public acceptance^4^. Early phytoremediation researches focused on hyperaccumulating plants because of their ability to concentrate high amounts of heavy metals in their tissues. However, hyperaccumulators often grow slowly and produce minimal biomass, and they generally only accumulate a specific toxic element from soil, which hinders their application in phytoremediation^5^. Tall fescue (*Festuca arundinacea Schreb*) is a major cool season forage and turf grass species grown in the temperate regions of the world. It was widely reported as a model species for rhizoremediation of organic contaminants due to its well-developed root system and broad adaptability to environment^6^. Early studies show that it has perfect resistance to heavy metal with high enrichment with various heavy metals including Cu, Cd, Pb, and Zn^6–11^, suggesting its remarkable prospects in phytoremediation.

The aim of this study was to evaluate toxicity of Cd in tall fescue roots and leaves and the response of tall fescue against Cd stress. To this purpose, 54 days old tall fescue were treated with different concentrations of cadmium ion (Cd^2+^) for two weeks, and Cd contents, leave lengths, chlorophyll contents, reactive oxygen species (ROS), malondialdehyde contents (MDA) were measured. Additionally, photosynthetic activities of the tall fescue leaves under Cd^2+^ stress or not were studied by fast chlorophyll fluorescence and slow chlorophyll fluorescence kinetic, and the results has been properly discussed.

## Materials and Methods

### Plant materials and Cd2+ treatment

A commercial type of tall fescue called “houndog 5” was seeded in plastic pots (7.5 cm in diameter and 8.5 cm deep) with gravel-perlite-peat (1:1:1, v/v/v) as medium. After germination, plants were kept in a greenhouse with a daily maximum/minimum temperature of 24/20 °C for 16 h photoperiod (300 µmol photons m^−2^s^−1^ PAR) allowing roots and shoots established^12^. The seedlings were watered daily, and fertilized twice a week with 1/2 Hoagland solution for 40 days. After that, the seedlings were transplanted to triangle bottles which were filled with 1/2 Hoagland solution, and cultivated for two weeks for acclamation. Cd^2+^ treatments were then carried out by addition of Cd^2+^ (CdCl_2_·4H_2_O, Sigma-Aldrich, St. Louis) to final concentrations of 0, 1, 5, 50, 150 mg/L Cd^2+^ for 2 weeks, and in this period culture medium was changed once every three days to maintain Cd^2+^ concentration.

### Biomass and chlorophyll content

Length of tall fescue shoots were recorded by vernier caliper^13^. Chlorophyll contents of tall fescue leaves were measured using SPAD-502 (Minolta, Osaka, Japan) according to the protocol provided by the instrument, which is a non-destructive, accurate, rapid and convenient instrument to quickly determine content of chlorophyll^14,15^.

### Cd^2+^ in tall fescue leaves and roots

Tall fescue roots and leaves were firstly washed with 10 mM EDTA for 3 times and 5 minutes for each time to remove Cd^2+^ adhered to their surface, then they were washed with deionized water and dried in oven at 60 °C to a constant weight. Then, dried leaves and roots were crushed by mortar and passed through a 100-mesh (0.15 mm) sieve. About 0.2 g of dried leaves and roots were put into digestion tanks with a mixture of HNO_3_ and H_2_O_2_ (4:1, v:v)^16^, then they were digested in a microwave digestion for 10 minutes. After suitable dilution with 1% HNO_3_, Cd^2+^ content in tall fescue leaves and roots were determined by flame atomic absorption analysis (AA-7001F, East & West Analytical Instrument). Cd^2+^ contents were expressed as mg g^−l^ plant dry weight (DW).

### Chlorophyll a fluorescence transient

In this study, all chlorophyll *a* fluorescence measurements were conducted by a PAM chlorophyll fluorometer (PAM 2500, Heinz Walz GmbH, Germany) with high time resolution (10 μs). For each test, measurements were repeated at least 4 times. After a dark-adaptation for 25 minutes, OJIP transients were induced by a red light of 3,000 photons μmol m^−1^s^−1^ provided by PAM 2500 through an array of light-emitting diodes. Chlorophyll *a* fluorescence emission induced by strong light pulses was then measured and digitized between 10 μs and 320 ms (Kautsky curve) and OJIP transients were analyzed using JIP-test as our previous study^12^.

### Slow chlorophyll fluorescence kinetics

Slow chlorophyll fluorescence kinetic was also performed using PAM 2500 as reported by our previous research with minimum modification^17^. Briefly, after 25 min dark adaptation, minimal fluorescence value (*F*o) was determined under far-red (FR) illumination (730 nm, 10 μmol photons m^−2^ s^−1^). Then a 0.8 s saturation pulse (3000 μmol photons m^−2^ s^−1^ PAR) was applied for recording of maximal fluorescence (*F*_m_). Subsequently, white actinic light (160 μmol photons m^−2^ s^−1^ PAR) was applied to induce photochemistry and reach steady-state condition. During the induction period, saturation pulses were applied during 30 s intervals to record light-adapted maximal fluorescence yield (*F*_m_′) to be used for quenching analysis. At the end of the induction period, immediately after switching off actinic light illumination, the minimal fluorescence at the light-adapted state (*F*_o_′) was recorded under FR illumination. Then relative parameters of slow chlorophyll fluorescence kinetics were read and recorded in the instrument.

### Determination of ROS and activity of ascorbate peroxidase

Hydrogen peroxide (H_2_O_2_) contents and superoxide radical (O^•^_2_^−^) were measured by spectrophotometry as our previous research^12^. 3,3′-diamino-benzidine (DAB) (Sigma-Aldrich, St. Louis) and nitroblue tetrazolium (NBT) (Sigma-Aldrich, St. Louis) were used to stain H_2_O_2_ and O^•^_2_^−^ respectively. Ascorbate peroxidase (APX) activitiy was determined by spectrophotometry, which uses H_2_O_2_ and ascorbate as substrate. Briefly, fresh leaves were ground in a mortar with liquid nitrogen, and homogenized in ice-cold sodium phosphate buffer (PBS, 50 mM, pH 7.8). The homogenate was centrifuged at 12,000 × g for 15 min at 4 °C, and supernatant was used as the crude extract for APX and protein content assay. Protein concentration was quantified by the Bradford method^18^, and APX activity was determined by APX test kit (Nanjing Jiancheng, China) according to the protocol provided by the manufacturer. The decrease in absorbance was recorded at 290 nm at an interval of 10 s up to 130 s. One unit APX activity was defined as in 1 mL reaction system catalysis 1 μmol ascorbate per minute per mg protein.

### Detection of cell death by PI staining

To assess cell membrane integrity of tall fescue roots with Cd^2+^ treatments or not, roots were immersed in 1 mg/mL propidium iodide (PI, Sigma-Aldrich, St. Louis) dissolved in distilled water. After washing, root samples were examined with a confocal laser scanning microscope (Leica TCS SP8, Germany) using an excitation wavelength of 546 nm.

### Lipid peroxidation

MDA content was determined by the method described by Hoeller^19^ with slight modification. In brief, fresh leaves were ground in a bowl chopper with liquid nitrogen, and then homogenized in ice-cold sodium phosphate buffer (50 mM, pH 7.8). The homogenate was then centrifuged at 12,000 g for 15 minutes at 4 °C. Then 1 mL enzyme solution was mixed with 2 mL of reaction solution containing 20% (v/v) trichloroacetic acid and 0.5% (v/v) thiobarbituric acid. The mixture was heated in a water bath at 100 °C for 30 minutes, then cooled to room temperature in ice-water bath and centrifuged at 10,000 × g for 10 minutes. Absorbance of the supernatant was measured at 532 and 600 nm. The content of MDA was calculated by subtracting the non-specific absorption at 600 nm from the absorption at 532 nm and calibrated using a extinction coefficient of 155 mM^−1^ cm^−1^. Lipid peroxidation was expressed as the MDA content in nmol per g fresh weight (FW).

### Statistical Analysis

Each experiment was repeated at least four times. Values were given as means ± SD. Statistical analyses were performed by analysis of variance (ANOVA), and means were separated using Duncan’s multiple range tests at a significant level of P < 0.05.

## Results

### Cd^2+^ accumulation in tall fescue roots and leaves

Cd^2+^ contents in tall fescue leaves and roots after 2 weeks’ exposure to different concentrations of Cd^2+^ were shown in Table 1. From the table, the highest Cd^2+^ contents in tall fescue roots and leaves were observed in the 150 mg/L Cd^2+^ treatment, and there were 25.73 and 0.73 mg/g (DW) respectively. Additionally, Cd^2+^ contents of roots and leaves increased with the increase of Cd^2+^ treatment concentration. Additionally, translocation factors (TF) were calculated as the ratio of Cd^2+^ contents in leaves to Cd^2+^ contents in roots, and under 50 mg/L and 150 mg/L Cd^2+^ treatments the values of TF were lower than those under 1 and 5 mg/L Cd^2+^ treatments.

**Table 1:** Cd^2+^ contents in tall fescue roots and leaves

Cd^2+^ treatment (mg/L)	Cd^2+^ content (mg/g DW)		TF
	Roots	Leaves	
0	nd	nd	nd
1	0.54 ± 0.11 d	0.026 ± 0.01 d	0.0481 a
5	1.57 ± 0.28 c	0.08 ± 0.02 c	0.0509 a
50	5.78 ± 2.4 b	0.18 ± 0.09 b	0.0311 b
150	25.73 ± 6.64 a	0.73 ± 0.06 a	0.0284 b

*nd, none detected; TF (translocation factor) = Con_leaves_/Con_roots_; DW, dry weight; data are given as mean ± SD of four independent experiments, and different letters indicate statistical difference significance at P < 0.05 among the treatments by Duncan’s multiple range tests.

### Effects of Cd^2+^ on growth and chlorophyll contents of tall fescue

**Figure 1:**
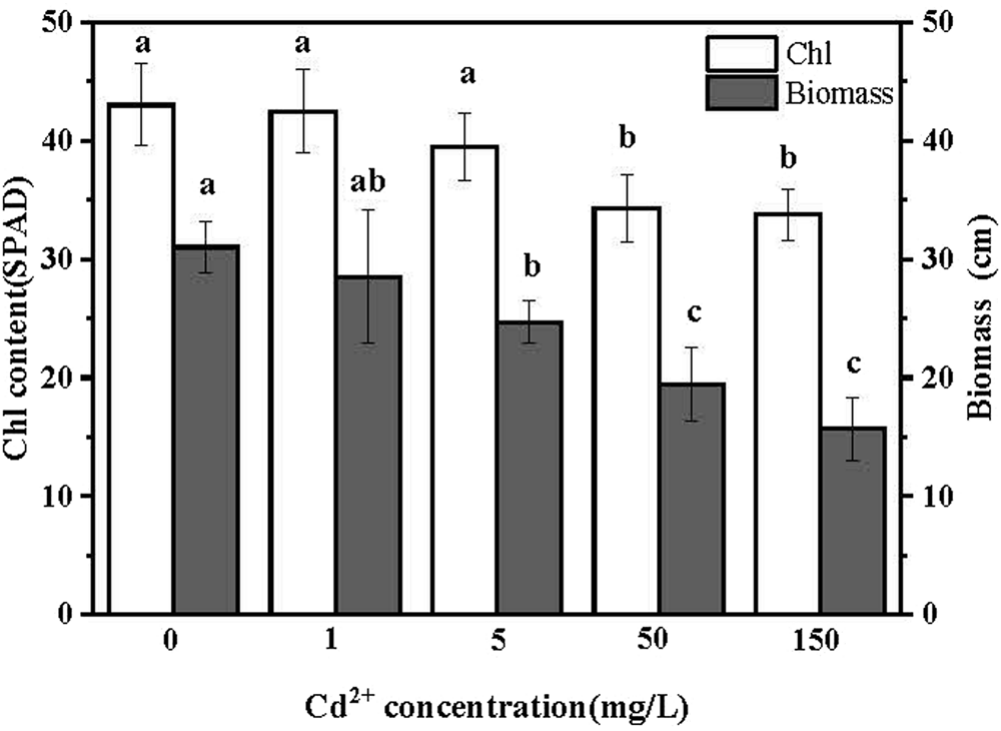
Effects of Cd^2+^ treatments on growth and chlorophyll contents of tall fescues. Values were given as means ± SD (n = 4). Means were separated using Duncan’s multiple range tests, and different letters indicates the significant difference at P < 0.05.

After two weeks exposure to Cd^2+^, toxic effects of Cd^2+^ in tall fescue biomass and chlorophyll content were shown in Fig. 1. Tall fescue biomass decreased with the increase of Cd^2+^ treatment concentration. Additionally, there was no significant difference between the values of tall fescue biomass under 0 and 1 mg/L Cd^2+^ treatments, and 50 mg/L and 150 mg/L Cd^2+^ treatments significant lower tall fescue biomass. The values of tall fescue chlorophyll content showed similar trend with the study of biomass. The chlorophyll contents of 50 and 150 mg/L Cd^2+^ treatments were significantly lower than that of the control group.

### Effects of Cd^2+^ in ROS production of tall fescue leaves and APX activity

**Figure 2:**
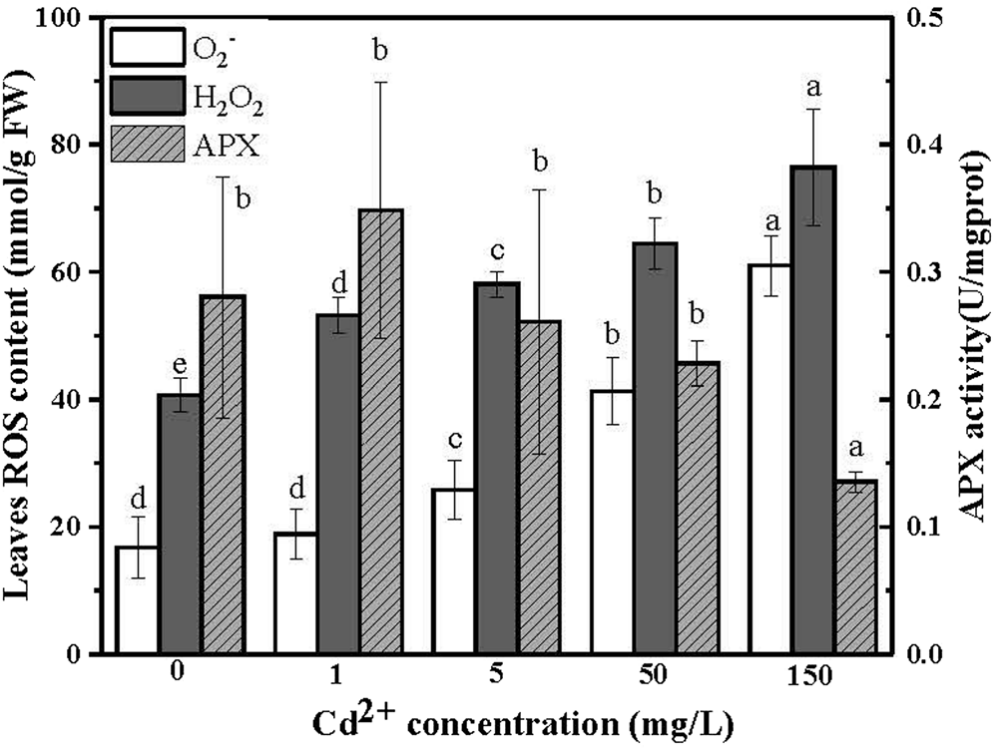
Effects of different Cd^2+^ treatments in ROS production and APX activity in tall fescue leaves. Values were given as means ± SD (n = 4). Means were separated using Duncan’s multiple range tests, and different letters indicate significant differences at P < 0.05.

Production of ROS contents responded to Cd^2+^ treatments were studied by the histological staining method and shown in Fig. 2. The highest ROS contents in tall fescue leaves was observed in the 150 mg/L Cd^2+^ treatment. The concentration of H_2_O_2_ in this treatment was 76.5 mmol/g (FW) and the concentration of O_2_^•−^ in this treatment was 60.1 mmol/g (FW). Additionally, contents of H_2_O_2_ in tall fescue leaves increased significantly with the increase of Cd^2+^ treatment concentration, and in 1 mg/L Cd^2+^ treatment the H_2_O_2_ content increased about 30.7% than that of the control. Contents of O_2_^•−^ also increased with the increase of Cd^2+^ treatment concentration. However, there was no significant difference between the contents of O_2_^•–^ of tall fescue subjected to 0 and 1 mg/L Cd^2+^ treatments. Additionally, APX activity principally decreased with the increase of Cd^2+^ treatment concentration, and APX activity of 150 mg/L Cd^2+^ treated leaf was the smallest than the other treatments.

### Effects of Cd^2+^ treatments in membrane integrity of root cells and MDA contents of leaves and roots

**Figure 3:**
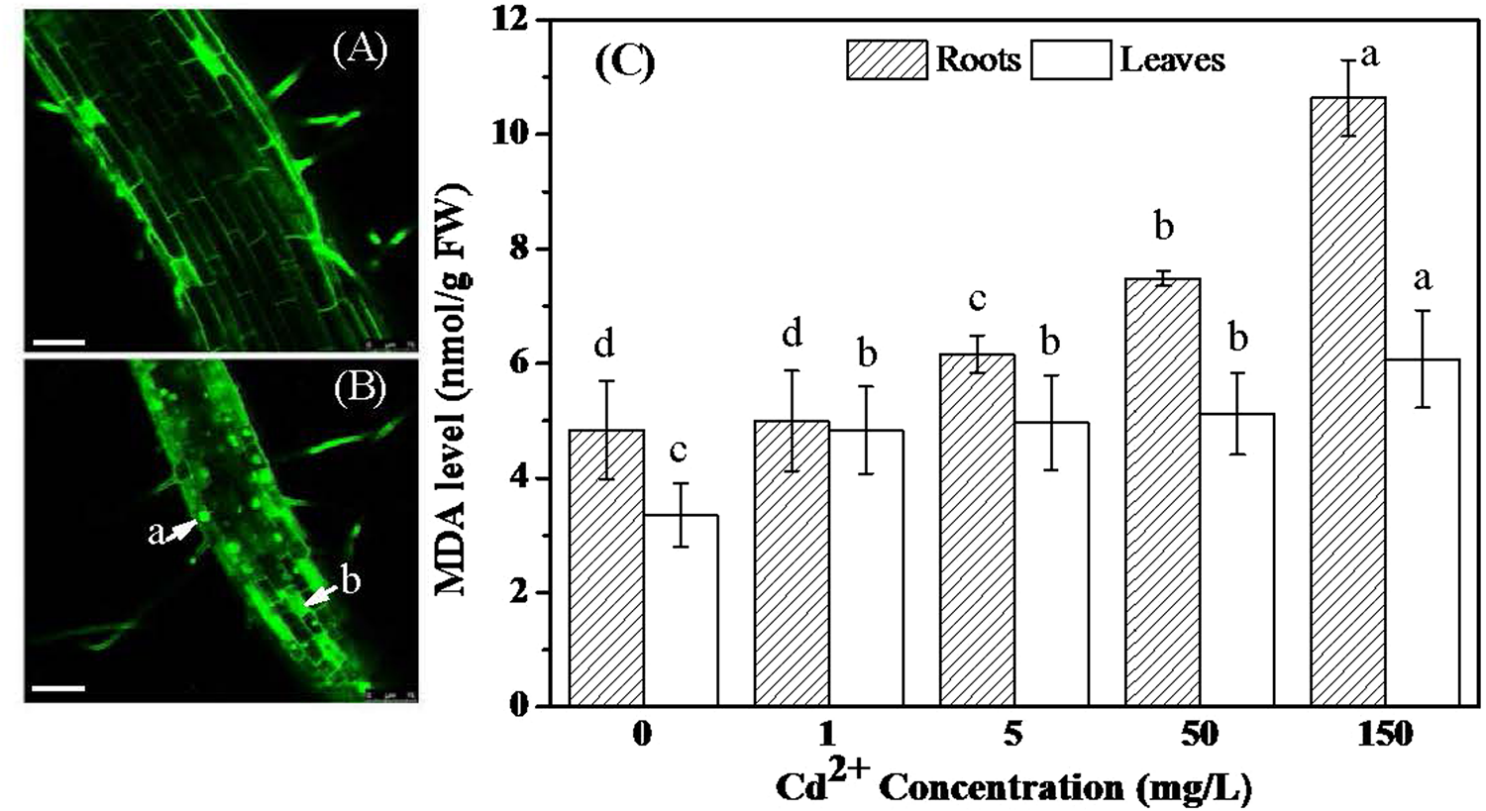
PI staining of tall fescue roots treated with 0 mg/L Cd^2+^ (**A**) and 5 mg/L Cd^2+^ (**B**), and effects of Cd^2+^ treatments on MDA contents of tall fescue roots and leaves (**C**). Values were given as means ± SD (n = 4), and means were separated using Duncan’s multiple range tests; different letters indicate significant difference at P < 0.05; bar = 75 μm.

Integrity of tall fescue root cells were studied by PI staining. Figure 3A and B showed PI staining figures of roots treated with 0 and 5 mg/L Cd^2+^, respectively. Arrow a and b indicated stained areas with the shape of round and square under the 5 mg/L Cd^2+^ treatment. In Fig. 3B we could find plenty of these kind of cells, while in Fig. 3A only three cells located on the margin of root were stained as this way. MDA contents of tall fescue leaves and roots after Cd^2+^ treatments were analyzed by spectrophotometry and shown in Fig. 3C. From the figure, MDA contents in roots treated with 5, 50 and 150 mg/L Cd^2+^ were higher than those treated with 0 and 1 mg/L Cd^2+^. Additionally, MDA contents in tall fescue leaves treated with Cd^2+^ were higher than that of the control, and MDA content of leaves treated with 150 mg/L Cd^2+^ was the highest.

### Effect of Cd^2+^ stress on JIP-test of tall fescue leaves

**Figure 4:**
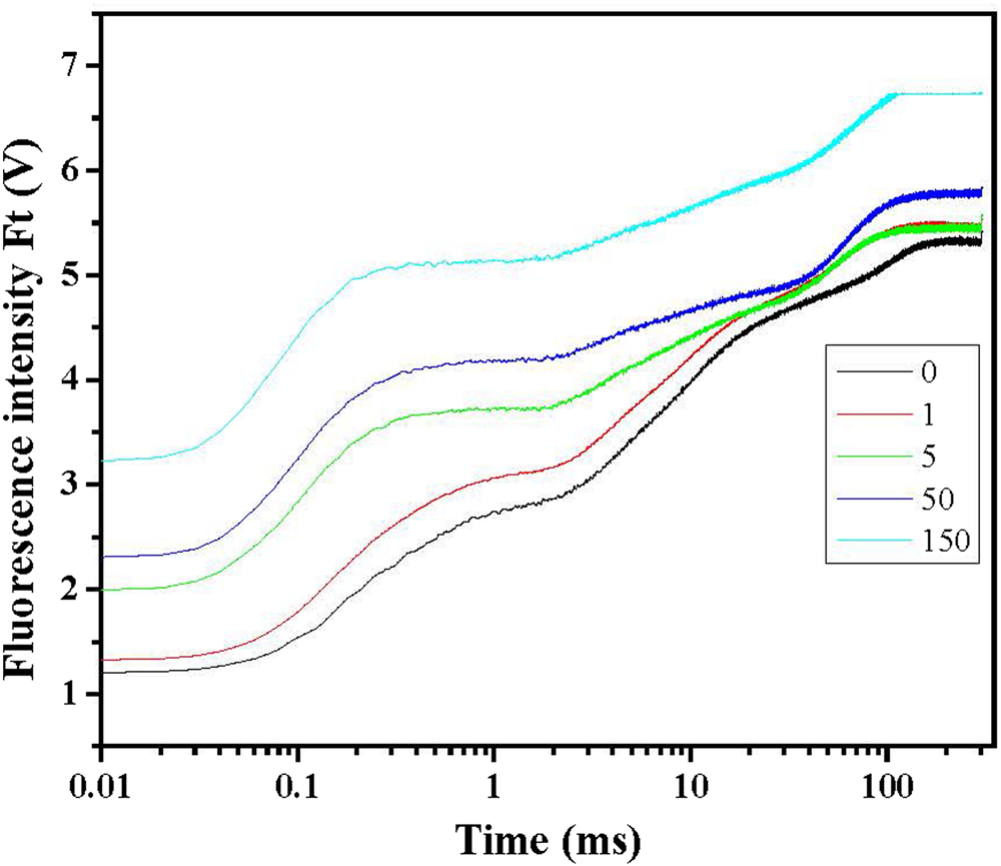
Effects of Cd^2+^ treatments on OJIP transient curves of tall fescue leaves. After a 25 min dark adaption period, the OJIP transients of tall fescue leaves were induced by a red light of 3000 μmol photons m^−2^ s^−1^ provided by PAM 2500 through an array of light-emitting diodes. Chlorophyll *a* fluorescence emissions were recorded by the instrument and most typical curves were shown here without normalization.

**Figure 5:**
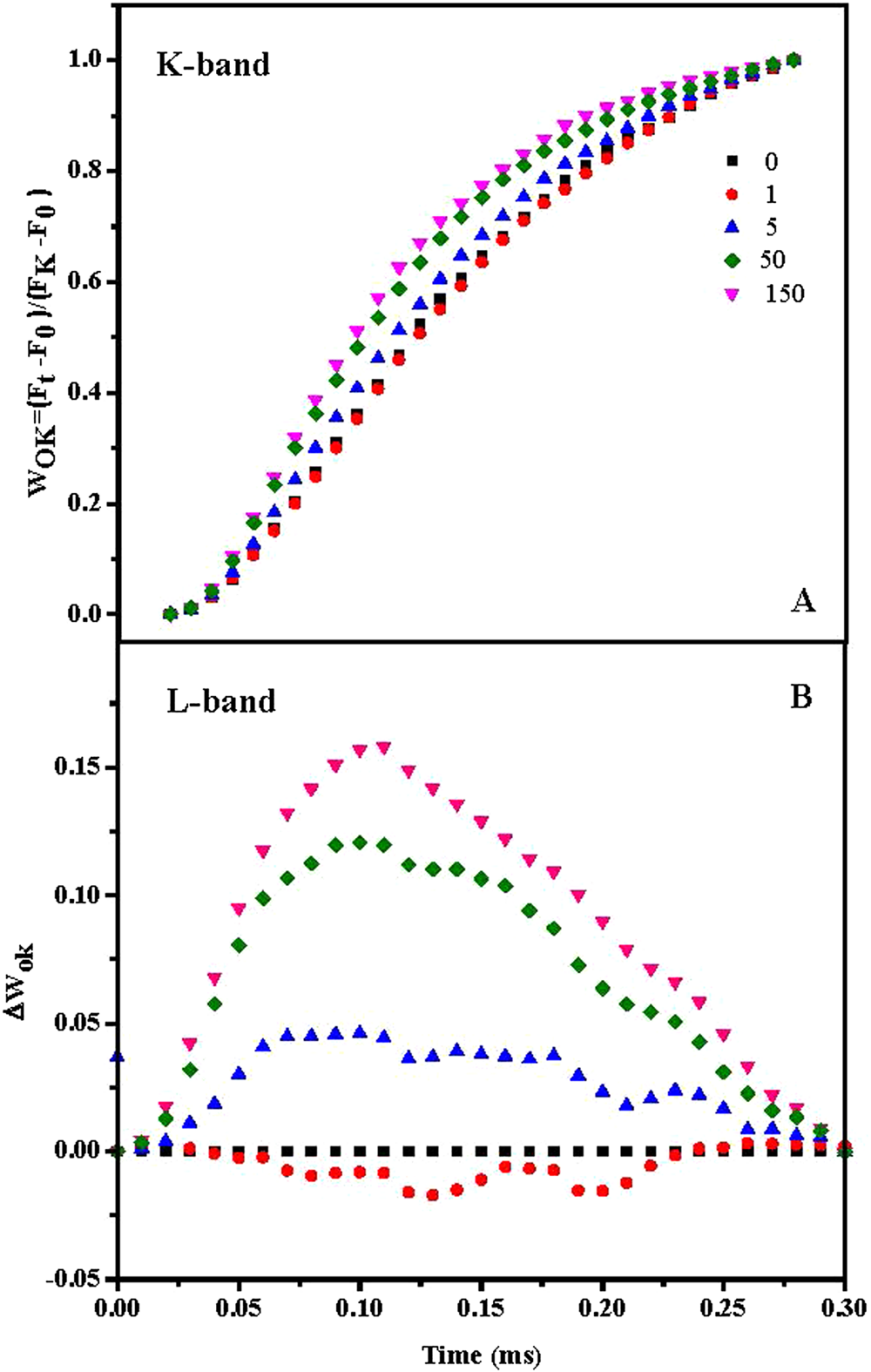
Effects of Cd^2+^ treatments on K-band and L-band of OJIP curves in tall fescue leaves. The difference of the Cd^2+^ treated samples to the control sample (0 mg/L Cd^2+^) (ΔV_t_), (A) between *F*_0_ and *F*_K_: W_OK_ = (*F*_t_ − *F*_0_)/(*F*_K_ − *F*_0_) and (B) the difference of the Cd^2+^ treated samples to the control sample (0 mg/L Cd^2+^) (ΔW_OK_).

As shown in Fig. 4, different Cd^2+^ treatments remarkably affect OJIP fluorescence transient curves of tall fescue leaves. Initial fluorescence of the OJIP curves increased with the increase of Cd^2+^ treatment concentration. Additionally, clear K-step of tall fescue leaves treated with 5, 50 and 150 mg/L Cd^2+^ were observed at 0.3 ms. To better understand toxic effect of Cd^2+^ on K step, K-band and L-band of the OJIP curves were studied and shown in Fig. 5. Figure 5A and B depict relative variable fluorescence between *F*_0_ and *F*_K_ (W_OK_) and differences of transients of Cd^2+^ treated samples (1, 5, 50, 150 mg/L Cd^2+^) minus control (0 mg/L Cd^2+^) (**Δ**W_OK_). In Fig. 5B, the sequence from upper to lower was tall fescue leaves treated with 150, 50, 5, 0 and 1 mg/L Cd^2+^.

The basic fluorescence parameters (Table 2) were extracted from the curves and subjected to the JIP-test. The JIP-test was used to further understand the energy flow and electron transport in PSII in this study, and the data of selected parameters and their definitions were shown in Table 3. Values of φ_P0_ and ψ_E0_ showed the same tendency that they were decreased with the increase of Cd^2+^ treatment concentration, while the values of φ_P0_ of tall fescue treated with 150 mg/L Cd^2+^ was significantly lower than the other treatments and the values of ψ_E0_ of tall fescue treated with 50 and 150 mg/L Cd^2+^ were significantly lower than the rest treatments. Meanwhile, the values of δ_R0_ showed an opposite tendency that they were generally increased with the increase of Cd^2+^ treatment concentration, and the value of δ_R0_ of 150 mg/L Cd^2+^ treatment was the highest. Additionally, values of PI_ABS_ and PI_total_ showed same tendency that they were decreased with the increase of Cd^2+^ treatment concentration, and values of PI_ABS_ and PI_total_ of the tall fescue treated with 150 mg/L Cd^2+^ were 62.7% and 39.5% smaller than that of the control, respectively.

**Table 2:** Basic photosynthetic parameters extracted from OJIP transient curves

Concentration (mg/L)	Photosynthetic parameters				
	*F*_0_	*F*_300_	*F*_J_	*F*_I_	*F*_M_
0	1.32 ± 0.11	2.62 ± 0.24	3.11 ± 0.25	4.46 ± 0.31	5.31 ± 0.21
1	1.34 ± 0.35	2.58 ± 0.42	3.11 ± 0.32	4.41 ± 0.40	5.12 ± 0.29
5	1.47 ± 0.35	2.88 ± 0.51	3.40 ± 0.38	5.08 ± 0.25	5.67 ± 0.35
50	2.28 ± 0.69	4.0 ± 0.74	4.10 ± 0.52	5.19 ± 0.58	6.08 ± 0.37
150	3.10 ± 0.88	4.21 ± 1.10	4.11 ± 0.93	5.41 ± 0.67	6.76 ± 0.41

*F_0_: fluorescence at 20 μs after the onset of actinic illumination; *F*_300_: fluorescence value at 300 μs; *F*_J_: fluorescence value at the J-step (2 ms) of OJIP; *F*_I_: fluorescence value at the I-step (30 ms) of OJIP; *F*_M_: fluorescence value at the peak of the OJIP test; Values are given as the means ± SD (n = 5–7).

**Table 3:** Photosynthetic parameters deduced by JIP-test of fluorescence transients.

Photosynthetic parameters	Cd^2+^ treatment concentration				
	0 mg/L	1 mg/L	5 mg/L	50 mg/L	150 mg/L
φP_0_	0.765 ± 0.01 a	0.758 ± 0.01 a	0.729 ± 0.02 a	0.722 ± 0.06 a	0.654 ± 0.05 b
ψE_0_	0.565 ± 0.04 a	0.564 ± 0.02 a	0.566 ± 0.03 a	0.529 ± 0.02 bc	0.504 ± 0.03 c
δR_0_	0.295 ± 0.03 c	0.302 ± 0.03 bc	0.340 ± 0.05 b	0.314 ± 0.04 bc	0.442 ± 0.05 a
ϒ_RC_	0.223 ± 0.02 a	0.215 ± 0.02 a	0.193 ± 0.03 b	0.198 ± 0.02 ab	0.164 ± 0.02 c
PI_ABS_	1.267 ± 0.38 a	1.134 ± 0.22 a	0.990 ± 0.31 ab	0.824 ± 0.36 b	0.473 ± 0.28 c
PI_total_	0.529 ± 0.07 a	0.491 ± 0.09 a	0.429 ± 0.07 a	0.321 ± 0.05 b	0.320 ± 0.07 b

*φ_Po_: maximum quantum yield for primary photochemistry, namely, F_V_/F_M_; Ψ_E0_: efficiency/probability with which a PSII-trapped electron is transferred from Q_A_ to Q_B_; δ_R0_: efficiency/probability with which an electron from Q_B_ is transferred to PSI acceptors; ϒ_RC_: probability that a PSII Chl molecule functions as RC; PI_ABS_: PI (potential) for energy conservation from exciton to the reduction of intersystem electron; PI_total_: PI (potential) for energy conservation from exciton to the reduction of PSI end acceptors. Subscript “0” indicates that the parameter refers to the onset of illumination. Values are given as the means ± SD (n = 5–7) and different letters indicate statistical difference significance at *P* < 0.05 among the treatments by Duncan’s multiple range tests.

### Effect of Cd^2+^ stress on slow chlorophyll fluorescence kinetics

To further understand the behavior of PSII under Cd^2+^ stress or not, slow chlorophyll fluorescence kinetics were also studied. As shown in Table 4, 1 mg/L Cd^2+^ treatment significantly lower Y(II) (actual quantum yield of photochemical energy conversion in PS II), and Y(II) decreased with the increase of Cd^2+^ treatment concentration. Additionally, the values of Y(NPQ) (the quantum yield of regulated non-photochemical energy loss in PS II) increased with the increase of Cd^2+^ treatment concentration. However, there were no significant difference between the values of Y(NO)(quantum yield of non-regulated non-photochemical energy loss in PS II) of tall fescue treated with Cd^2+^ or not. Meanwhile, qP (coefficients estimating the fraction of open PS II reaction centers based on a puddle model) and qL (coefficients estimating the fraction of open PS II reaction centers based on a lake model) reflect fraction of open reaction center, and the difference is that qP is based on the puddle antenna model and qL is based on the lake antenna model^20^. In our study, both of them were decreased with the increase of Cd^2+^ treatment concentration. Moreover, the values of ETR (electron transfer rate) decreased with the increase of Cd^2+^ treatment concentration.

**Table 4:** Photosynthetic parameters deduced by slow Chl fluorescence kinetics

**Photosynthetic parameters**	**Cd** ^**2+**^ **treatment concentration**				
	0 mg/L	1 mg/L	5 mg/L	50 mg/L	150 mg/L
Y(II)	0.610 ± 0.02 a	0.532 ± 0.03 b	0.538 ± 0.04 b	0.538 ± 0.03 b	0.445 ± 0.02 c
Y(NPQ)	0.103 ± 0.02 c	0.184 ± 0.03 b	0.184 ± 0.04 b	0.185 ± 0.04 b	0.263 ± 0.02 a
Y(NO)	0.286 ± 0.01 a	0.284 ± 0.08 a	0.277 ± 0.01 a	0.277 ± 0.01 a	0.292 ± 0.01 a
NPQ	0.360 ± 0.06 c	0.647 ± 0.08 b	0.668 ± 0.15 b	0.672 ± 0.17 b	0.905 ± 0.09 a
qN	0.336 ± 0.04 c	0.489 ± 0.04 b	0.494 ± 0.06 b	0.492 ± 0.07 b	0.585 ± 0.02 a
qP	0.863 ± 0.02 a	0.794 ± 0.03 b	0.802 ± 0.03 b	0.796 ± 0.03 b	0.697 ± 0.02 c
qL	0.649 ± 0.03 a	0.561 ± 0.03 b	0.573 ± 0.04 b	0.559 ± 0.03 b	0.455 ± 0.03 c
ETR	28.7 ± 1.0 a	25.2 ± 1.5 b	25.3 ± 1.8 b	25.3 ± 1.6 b	20.8 ± 0.8 c

*Y(II), quantum yield of photochemical energy conversion in PS II; Y(NPQ), the quantum yield of regulated non-photochemical energy loss in PS II; Y(NO), quantum yield of non-regulated non-photochemical energy loss in PS II; NPQ, non-photochemical quenching parameter; qN, coefficients of non-photochemical quenching; qP, coefficients estimating the fraction of open PS II reaction centers based on a puddle model; qL, coefficients estimating the fraction of open PS II reaction centers based on a lake model; ETR, electron transfer rate (μmol electrons m^−2^ s^−1^). Values are given as the means ± SD (n = 5–7) and different letters indicate statistical difference significance at *P* < 0.05 among the treatments by Duncan’s multiple range tests.

## Discussion

Photosynthates are the main source for biomass production^21^. Toxicity of Cd^2+^ significantly affect chlorophyll contents of tall fescue leaves and finally affect growth of tall fescue as shown in Fig. 1. In our study, compared to the control, 5 mg/L Cd^2+^ treatment for 14 days decreased the biomass of tall fescue for 20.4%, which exhibited comparable high Cd^2+^ tolerance than Indian mustard (*Brassica juncea* L.). Indian mustard is well known for its superior tolerance to Cd^2+^ toxicity^22^, and Vatehová reported that after 7 days treatment with 4.48 mg/L Cd^2+^ the shoots biomass of Indian mustard decreased about 25.6%^23^. There are some studies carried out with soilless medium and also concern the amount of Cd^2+^ absorbed by the tall fescue^10,24,25^. Due to the differences in the purpose of the study, we measured Cd^2+^ contents in roots with an EDTA wash step before digestion to exclude Cd^2+^ adsorbed on the surface of the roots. In this case, tall fescue still showed high absorbing ability, and in the 150 mg/L Cd^2+^ treatment, tall fescue absorbed 25.73 mg/g (DW) Cd^2+^ in their roots in 14 days.

In plants, Cd^2+^ could cause production of superoxide radicals and finally induce oxidative stress^26^. In this study, concentrations of H_2_O_2_ and O_2_^.−^ increased significantly with the increase of Cd^2+^ treatment concentration (Fig. 2). In plant cells, electron transport processes in chloroplasts and mitochondria are the potential source of ROS^3^, and plants develop their own regulation mechanism against oxidative stress, such as antioxidant relative enzyme^27^. However, presence of Cd^2+^ in plants could induce ROS formation^28^ by inhibition of ROS degradation enzyme (Fig. 2)^29^. In this case, the balance between ROS production and ROS scavenging mechanisms has been disturbed and lead to damage to proteins, lipids and carbohydrates^30^.

MDA is an oxidized product of membrane lipids and accumulates when plants are exposed to oxidative stresses^10^. Therefore, the concentration of MDA in plants is commonly considered as a general indicator of lipid peroxidation as well as oxidant stress level. Lipid peroxidation is closely relevant to ROS level, because H_2_O_2_ and O_2_^.−^ can initiate lipid peroxidation that results in formation of lipid hydroperoxide (LOOH)^31,32^. In this study, the MDA contents showed the tendency that they were increased with the increase of Cd^2+^ treatment concentration in both tall fescue roots and leaves. However, under the same treatment condition, the MDA contents in tall fescue leaves were generally lower than those in tall fescue roots, which may because tall fescue roots in this study were subjected to the toxicity of the Cd^2+^ directly and only few amounts of Cd^2+^ were transferred to leaves and lead to a relative lower damage to tall fescue leaves.

In this study, roots are primary contact site for Cd^2+^ in medium. Lower to 5 mg/L Cd^2+^ treatment induced significant difference of the MDA content in tall fescue roots between the control and Cd^2+^ treatments. Toxic effect of Cd^2+^ in tall fescue roots were further studied by PI staining method. PI could bind to pectin^33^ and DNA^34^ by intercalating between the bases, and it is used to stain cell wall as well as dead cells. A PI-positive nucleus is a strong indication of loss of membrane integrity due to its membrane-impermeable property, which is generally excluded from viable cells^35^. The arrow a and b in Fig. 3B indicate two typical PI staining cells with the shape of square and round. The round cells in the figure mean that they had lost membrane integrity, and the square cells mean the cell nuclei were disintegrated and cells were undergoing necrosis and apoptosis. Comparing to the control, a large number of cells in tall fescue roots treated with 5 mg/L Cd^2+^ lost their membrane integrity and underwent necrosis and apoptosis. The Cd^2+^ induced necrosis and apoptosis has been reported in plants^36,37^. In this study, Cd^2+^ induced ROS production may trigger the apoptosis of root cells, as a signaling event, and lead to cell necrosis as shown in Fig. 3 ^26,38,39^.

The photosynthesis activities of tall fescue subjected to different Cd^2+^ treatments had been studied by fluorescence transient curves and shown in Fig. 4. From the figure, increased Cd^2+^ treatments increased initial fluorescence (*F*_0_, equal to *F*_20_ or *F*_50_). After dark adaption, primary quinone acceptors in PSII are in oxidized state, and *F*_0_ could be obtained by fluorescence emission from PSII induced by weak illumination. It refers to concepts of open, close and inactivation of reaction center (RC) in PSII^40^. In our study, the higher value of *F*_0_ induced by Cd^2+^ treatment could be attribute to inactivation of RC. It has been reported that Cd^2+^ could exchange Mg^2+^ in Light Harvest Complex II (LHCII) and then disrupt its function^41,42^. Cd^2+^ exposure would result in disorganization of trimer-forming monomers of LHCII and lead to diminished LHCII aggregation complex^43^, and Lhcb1.1 isomers of LHCII was highly sensitive to Cd^2+^ stress^44^. Additionally, inactivation of RC is also proved by the study of ϒ_R_. ϒ_R_ reflects probability that a PSII chlorophyll molecule functions as RC, and ϒ_RC_ in this study showed a tendency that they decreased with the increase of Cd^2+^ treatment concentration, and the value of ϒ_RC_ of 150 mg/L Cd^2+^ treatment was significant lower than the rest treatments (Table 3). Moreover, qP and qL decreased with the increase of Cd^2+^ treatment concentration (Table 4), which suggests that under Cd^2+^ stress, RC in tall fescue were partially closed.

Clear K-step of OJIP curves of tall fescue treated with 5, 50 and 150 mg/L Cd^2+^ were observed at 0.3 ms, which suggests that higher than 5 mg/L Cd^2+^ treatment could lead to inhibited electron transport from OEC to tyrosine residue (Yz, D1-Tyr161). Figure 5 explores K-band and L-band of OJIP curves of tall fescue treated with Cd^2+^ and gave more details about this step. From the figure, higher than 5 mg/L Cd^2+^ treatments increased amplitude of L-band, which is in accord with the finding about the appearance of the K-step at 0.3 ms. Inhibited electron transport from OEC to Yz may attribute to, firstly, damage of D1 protein induced by ROS. PsbA encoded D1 protein accepts electron from OEC and toxic nature of the water-oxidation reaction defines the property of rapid turnover of D1 protein. Highly reactive O_2_^.−^ produced in water-oxidation reaction is extremely destructive to D1 protein^45^, and Cd^2+^ induced ROS production (Fig. 2) may enhance susceptibility of this protein. Secondly, Cd^2+^ could replace Ca^2+^ in OEC with high affinity and inhibit activity of OEC. A high-resolution structural analysis reveals that OEC contains five oxygen in addition to four Mn and one Ca atoms, forming a Mn_4_CaO_5_-cluster^46^. It has been reported that Cd^2+^ could competitively bind to essential site belong to Ca^2+^ and lead to inhibition of photosynthetic oxygen evolution, and finally inhibited electron transport from OEC to Yz^47–49^.

OJIP transient data were analyzed using JIP-test as our previous research^12^, which was developed by Strasser and based on the theory of energy fluxes in bio-membranes^40,50,51^. Electron transport in photosystem was divided to three major steps, namely, trapping of photon, electron transport after quinone A (Q_A_^−^) to intersystem electron acceptors and reduction of end acceptors^52^. φ_Po_ depicts efficiency of trapping photon which mainly refers to electron donor side in PSII. In our study, the treatments with high content of Cd^2+^ significantly lower the values of φ_Po_, which is in accord with the finding we have discussed above. In electron donor side, electron transport efficiency from OEC to Yz were inhibited by Cd^2+^, which may be due to the increase of ROS contents and the replacement of Ca^2+^ located in the core of OEC. Additionally, we found that δ_R0_ principally increased with the increase of Cd^2+^ treatment concentration. It means that electron transport efficiency from quinone B (Q_B_) to the PSI acceptors increased under high Cd^2+^ treatments, which may be an important response for plants against Cd^2+^ toxicity. It has been reported that antenna proteins of PSI and Light Harvest Complex 1 (LHC1) reduced significantly in *Spinacia oleracea* L. under Cd^2+^ stress^44^. However, it is still not clear how reduced antenna proteins and LHC1 affect electron transport efficiency from Q_B_ to the PSI acceptors. Although we could not find relative research about effect of Cd^2+^ in electron transport from Q_B_ to PSI electron acceptor, Schottler reported that mobile carrier plastocyanin (PC) exhibited a nearly linear relationship with PSI flux rate. This finding suggests that PC may play an important role in the response of PSI against Cd^2+^ toxicity and it needs our further study.

Additionally, in this study both PI_ABS_ and PI_Total_ decreased with the increase of Cd^2+^ treatment concentration, which is in accord with the result obtained by slow fluorescence kinetics study (Table 4), which showed that ETR decreased with the increase of Cd^2+^ treatment concentration. Additionally, it is also in accord with the result reported by Zurk^53^. That could principally be attributed to inhibited activities in the electron donor side. Although the electron transport efficiency from Q_B_ to the PSI acceptors increased with the increase of the Cd^2+^ treatment concentration, electrons arrived to Q_B_ still only take a small portion compared to electrons absorbed by PSII antenna pigments.

The effect of Cd^2+^ on the mode of the yields for dissipative processes for energy absorbed by tall fescue PS II were studied and shown in Table 4. In any case, the sum of Y(II), Y(NPQ) and Y(NO) is unity. In Table 4, significant difference between Y(II) of tall fescue treated with 0 and 1 mg/L Cd^2+^ is obtained, and it proves that this parameter is more sensitive than φ_Po_. which suggest that it could be a better stress indicator than φ_Po_, even the measurement of slow kinetics is much more time costly_._ Additionally, Y(NPQ) increased with the decrease of Y(II), and there was no significant difference between the values of Y(NO) of tall fescue treated with Cd^2+^ or not. This result suggests that under Cd^2+^ stress more absorbed energy flux was dissipated via regulated non-photochemical quenching in PSII to avoid overreduction of electron transport chain in plants under stress condition^17^, which represents an important photoprotective mechanism in plants^54^.

## Conclusion

In this study, toxic effects of Cd^2+^ in tall fescue and the response of tall fescue against Cd^2+^ toxicity were studied. Tall fescue exhibited high tolerance and bioconcentration to Cd^2+^. High content of Cd^2+^ leaded to decreased biomass and chlorophyll contents, production of ROS and lipid peroxidation, as well as necrosis and apoptosis in tall fescue roots. Additionally, in PSII the electron transport from OEC to Yz residue in D1 protein was inhibited under high Cd^2+^ treatments, which may be due to the Cd^2+^ induced ROS production and the replacement of Ca^2+^ in the core of OEC. The electron transport efficiency from Q_B_ to PSI acceptors increased under high Cd^2+^ treatments, which may be an important response of tall fescue against Cd^2+^ toxicity and needs our further study.
